# Supplementary material for: Transcriptome Dynamics during Spike Differentiation of Wheat Reveal Amazing Changes in Cell Wall Metabolic Regulators
Source: Int J Mol Sci. 2023 Jul 19;24(14):11666. doi: 10.3390/ijms241411666 (PMC10380499; doi:10.3390/ijms241411666)
Supplement: Supplementary file 1 [file ijms-24-11666-s001.zip › Supplementary_Figures Several figures cited in the article.pdf]

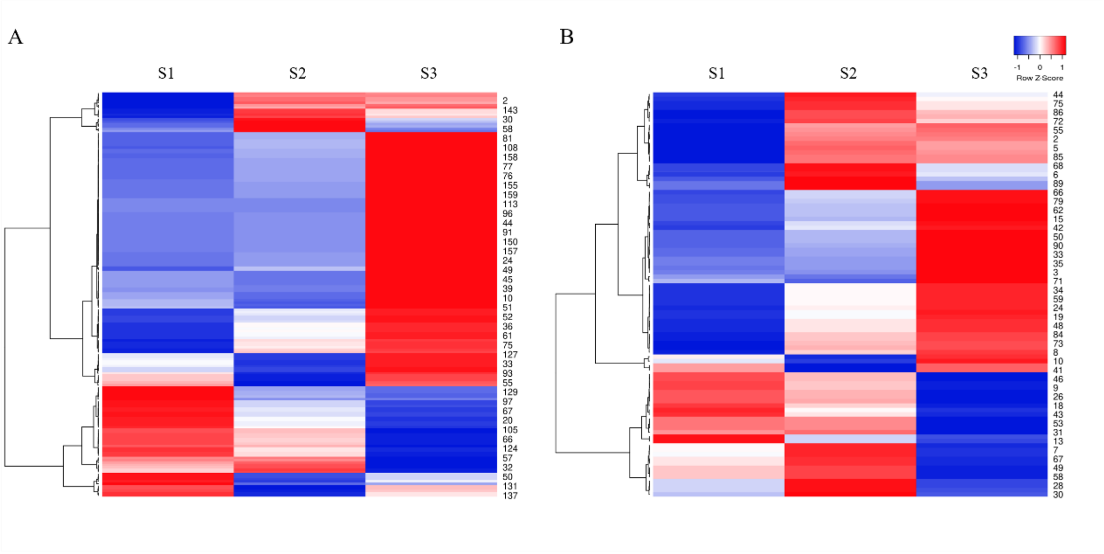

Supplementary Figure S1. Heat map of expression patterns of acyltransferase (AT) and peroxidase (POD). Data were obtained using the $\log_2$fragments per kilobase of exon per million fragments mapped (FPKM) of each gene. Red and blue represent up- and downregulated genes, respectively. A, acyltransferase; B, peroxidase.

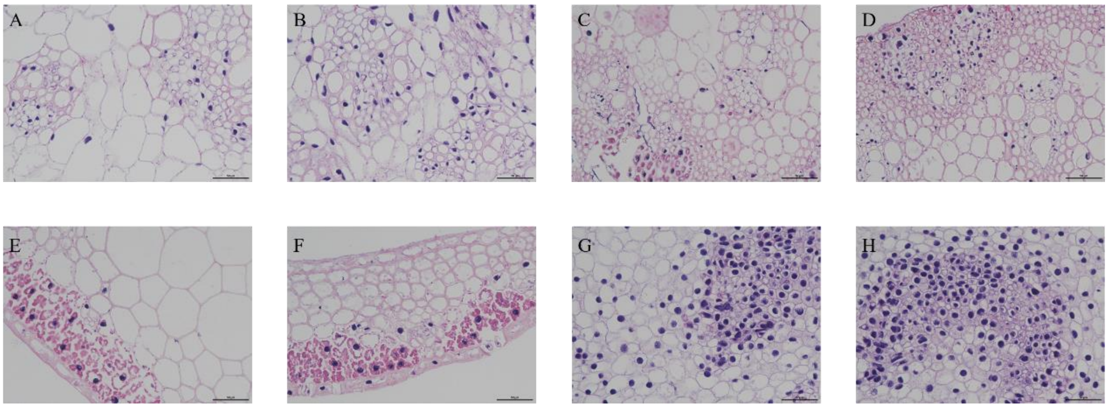

Supplementary Figure S2. Microscopic observation of WT and *TraesCS7A02G426700*-overexpression wheat. Microscopic observation was conducted on wheat spike-stalk with transgenic plants (A) and wild type plants (B). Microscopic observation was performed on the stem under the spike of transgenic wheat plants (C) and wild type plants (D). Microscopic observation was performed on floret with transgenic wheat plants (E) and wild type plants (F). Microscopic observation of the pre pollination ovaries of transgenic wheat plants (G) and wild-type plants (H).

Scale bar = 50 μm.
